# Efficacy of metformin in pregnant obese women: a randomised controlled trial

**DOI:** 10.1136/bmjopen-2014-006854

**Published:** 2015-01-14

**Authors:** Carolyn A Chiswick, Rebecca M Reynolds, Fiona C Denison, Sonia A Whyte, Amanda J Drake, David E Newby, Brian R Walker, Shareen Forbes, Gordon D Murray, Siobhan Quenby, Susan Wray, Jane E Norman

**Affiliations:** 1 Tommy's Centre for Fetal and Maternal Health, Medical Research Council Centre for Reproductive Health, Queen's Medical Research Institute, The University of Edinburgh, Edinburgh, UK; 2 Endocrinology Unit, University/BHF Centre for Cardiovascular Science, Queen's Medical Research Institute, University of Edinburgh, Edinburgh, UK; 3 University/BHF Centre for Cardiovascular Science, Queen's Medical Research Institute, University of Edinburgh, Edinburgh, UK; 4 Centre for Population Health Sciences, University of Edinburgh, Edinburgh, UK; 5 Department of Reproductive Health, Clinical Science Research Institute, Warwick Medical School, University Hospital, Coventry, UK; 6 Department of Cellular and Molecular Physiology, Institute of Translational Medicine, University of Liverpool, Liverpool, UK

## Abstract

**Introduction:**

Increasing evidence suggests obesity has its origins prior to birth. There is clear correlation between maternal obesity, high birthweight and offspring risk of obesity in later life. It is also clear that women who are obese during pregnancy are at greater risk of adverse outcomes, including gestational diabetes and stillbirth. The mechanism(s) by which obesity causes these problems is unknown, although hyperglycaemia and insulin resistance are strongly implicated. We present a protocol for a study to test the hypothesis that metformin will improve insulin sensitivity in obese pregnant women, thereby reducing the incidence of high birthweight babies and other pregnancy complications.

**Methods and analysis:**

The Efficacy of Metformin in Pregnant Obese Women, a Randomised controlled (EMPOWaR) trial is a double-masked randomised placebo-controlled trial to determine whether metformin given to obese (body mass index >30 kg/m^2^) pregnant women from 16 weeks’ gestation until delivery reduces the incidence of high birthweight babies. A secondary aim is to test the mechanism(s) of any effect. Obese women with a singleton pregnancy and normal glucose tolerance will be recruited prior to 16 weeks’ gestation and prescribed study medication, metformin or placebo, to be taken until delivery. Further study visits will occur at 28 and 36 weeks’ gestation for glucose tolerance testing and to record anthropometric measurements. Birth weight and other measurements will be recorded at time of delivery. Anthropometry of mother and baby will be performed at 3 months postdelivery. As of January 2014, 449 women had been randomised across the UK.

**Ethics and dissemination:**

The study will be conducted in accordance with the principles of Good Clinical Practice. A favourable ethical opinion was obtained from Scotland A Research Ethics Committee, reference number 10/MRE00/12. Results will be disseminated at conferences and published in peer-reviewed journals.

**Trial registration number:**

ISRCTN51279843.

**Strengths and limitations of this study**

- This is the first multicenter, double-masked, randomised controlled trial to examine the effect of metformin in obese pregnant women without diabetes.
- We will use a recognised surrogate (birthweight centile) as a marker of future life risk of obesity in the offspring.
- Follow-up of the offspring is limited to the early postnatal phase.

## Background, including rationale and any previous systematic reviews

Rates of obesity, as defined by a body mass index (BMI) greater than 30 kg/m^2^, are rising alarmingly in the UK and throughout the world. Around 27% of adults in Scotland are now obese and 32% of Scottish children have a weight outwith a healthy range for their age.^1^ Obesity rates in England^2^ and the USA^3^ are similar. The increase in average BMI during pregnancy over the past 20 years has been well documented.^4^ Rates of maternal obesity in women booking for antenatal care in the UK are now around 20%. There has also been a substantial increase in mean birth weight and in the incidence of being born large for gestational age over the past few decades.^5^ The secular rise in maternal weight at delivery appears to be the factor most strongly correlated with the increase in birth weight.^5^ ^6^ Positive correlations have also been shown between birth weight and both maternal pregravid weight^7–9^ and weight gain in pregnancy,^10^ with odds of high birth weight some 2.4 times greater in morbidly obese compared with lean women.^11^

This increase in mean birth weight is of concern because it is linked to an increased likelihood of child and adult obesity. A systematic review showed a positive correlation between birth weight and obesity during adulthood.^12^ The association between high birth weight and later life obesity is also supported by two large epidemiological studies^13^ ^14^ both of which found obesity to be a long-term correlate of high birth weight. A subsequent prospective study found that children who were large for gestational age at birth and exposed to an intrauterine environment of maternal obesity are also at increased risk of developing metabolic syndrome.^15^ Our own recent work suggests that offspring of obese pregnant women are at increased risk of premature death in adulthood.^16^ Thus, the increase in rates of maternal obesity are setting up a vicious cycle, leading to increased birth weight and increased rates of child and adult obesity, contributing to an epidemic of obesity which has become one of the most significant contributors to global ill health.

Women who are obese during pregnancy are also themselves at greater risk of a number of adverse outcomes, including gestational diabetes,^17^ pre-eclampsia^18^ and stillbirth.^19^ ^20^ The mechanism(s) by which maternal obesity increases pregnancy and peripartum complications are unclear, but there are likely several candidate mechanisms including hyperglycaemia and increased insulin resistance.

Clearly an intervention is urgently required. Lifestyle interventions (eg, diet and exercise) are a logical approach and are currently being trialled, for example, UK Pregnancies Better Eating and Activity Trial (UPBEAT), Poston (ISRCTN89971375) although studies hitherto have shown no evidence of benefit.^21–23^ A recent meta-analysis of dietary and lifestyle interventions suggests dietary interventions may be of some benefit over physical activity or mixed interventions in reducing maternal weight gain. However, there was no effect on birth weight, the incidence of large for gestational age babies and clinically important adverse outcomes.^24^

In this study, we test the hypothesis that the insulin-sensitising agent metformin reduces birthweight centile in the offspring of obese pregnant women. The principal action of metformin includes improvement of insulin resistance in the liver and skeletal muscle, along with improved endothelial function, increased peripheral uptake of glucose, improved lipid profile, redistribution of fat from visceral to subcutaneous depots and antioxidant effects,^25^ all of which are likely to contribute to its clinical efficacy in reducing adverse outcomes in obese pregnant women.

Considerable evidence implicates insulin resistance or hyperglycaemia as the mechanism by which maternal obesity causes excessive neonatal birth weight. While modest insulin resistance is physiological in pregnancy and generates maternal glucose, free fatty acids and amino acids as substrates for fetal growth, obese pregnant women are more insulin resistant than their lean counterparts^6^ leading to a further amplification of nutrient availability with consequent excessive fetal growth. There is a strong correlation between insulin resistance in late gestation and both birth weight and fat-free mass at birth.^26^ The Hyperglycaemia and Adverse Pregnancy Outcomes (HAPO) trial^27^ confirms that there is a linear relationship between hyperglycaemia and birth weight, even at glucose levels not usually considered abnormal during pregnancy. The Australian Carbohydrate Intolerance Study in Pregnant Women (ACHOIS)^28^ confirms that treating hyperglycaemia can substantially reduce both the incidence of large for gestational age babies and other perinatal complications. Thus, metformin, by reducing insulin resistance, could have a major impact in reducing excess birth weight in obese pregnant women.

This study is timely because of emerging evidence about the safety and efficacy of metformin. It is a licensed first-line therapy for the treatment of type 2 diabetes and is endorsed by National Institute for Health and Care Excellence (NICE) as a treatment for gestational diabetes.^29^

There are few previous studies testing the effects of metformin in pregnant women in the absence of diabetes. A small randomised study of 40 pregnant women with a history of polycystic ovarian syndrome (PCOS), a condition often accompanied by insulin resistance, showed that metformin reduced predefined pregnancy complications from 32% in the placebo group (n=22) to 0% in the metformin group (n=18; p=0.01).^30^ However, a larger study by the same group, which randomised 274 women with PCOS to either metformin or placebo throughout pregnancy, showed no significant differences in the prevalence of preterm birth, pre-eclampsia and gestational diabetes.^31^ There was also no difference in birth weights of the babies in the two groups. However, the mean BMI of the participants in these studies was less than 30 kg/m^2^. In the Metformin in Gestational diabetes (MiG) study, which compared the effect of metformin versus insulin in women with gestational diabetes,^32^ there were no differences in birth weight between the offspring. However, weight gain in pregnancy, a known additional driver of birth weight,^7^ was lower in the metformin group (difference of 1.6 kg, p<0.001). By 2 years of age, offspring of the participants randomised to metformin had greater subcutaneous fat, but overall body fat was the same as in children whose mothers were treated with insulin alone, suggesting that the ‘metformin offspring’ have smaller deposits of visceral, metabolically active fat.^33^ Further follow-up is required to examine whether these findings persist into later life and whether children exposed to metformin will be more insulin sensitive.

Importantly, if metformin is to be effective in reducing high birth weight in obese women, it should not increase the proportion of babies with low birth weight. The studies aforementioned, and another small study, on women with a mean BMI less than 30 kg/m^2^ are reassuring on this point.^30^ ^31^ ^34^

## Hypothesis

We hypothesise that metformin administered to obese women during pregnancy will reduce birthweight centile in their babies and consequently their future life risk of obesity and metabolic syndrome.

## Aim

To determine if metformin administered to obese women during pregnancy reduces birthweight centile in their babies, using birth weight as a surrogate marker of the future life risk of obesity and metabolic syndrome in the offspring.

## Objectives

### Primary objective

To determine the efficacy of metformin (up to 2500 mg daily), given to obese pregnant women from between 12 and 16 weeks’ gestation until delivery, in reducing gestational age-adjusted and sex-adjusted birthweight centile of the baby.

### Secondary objectives

- To determine the pattern of association between insulin resistance and adverse pregnancy outcomes, including incidence of pregnancy-induced hypertension, pre-eclampsia, caesarean section, postpartum haemorrhage, maternal weight gain during pregnancy and incidence of the baby's admission to the neonatal unit.
- To determine the effect of metformin on maternal body composition.
- To determine the effect of metformin on neonatal body composition.
- To determine the effect of metformin on maternal inflammatory and metabolic variables (measured at 28 and 36 weeks’ gestation) and on neonatal inflammatory variables (measured in cord blood at birth).
- To confirm that metformin does not increase the rate of babies with a low birthweight centile.
- To determine the efficacy (as opposed to the effectiveness) of metformin when analysis is restricted to those with pharmacological circulating levels of drug.

A series of nested substudies will also be performed with the following objectives:

- To determine the effect of metformin on maternal cortisol levels in obese pregnant women.
- To determine the effect of metformin in hepatic and peripheral insulin sensitivity at 36 weeks’ gestation in obese pregnant women.
- To determine the effect of metformin on endothelium-dependent flow-mediated dilatation in obese pregnant women.
- To determine the effect of metformin on maternal subcutaneous and visceral adipose tissue deposition and hepatic and skeletal muscle ectopic fat deposition during pregnancy.
- To determine the effect of metformin on fetal liver volume and subcutaneous fat deposition.
- To determine the effect of metformin on myometrial contractility and myometrial glycogen storage in obese pregnant women.
- To determine the effect of metformin on placental glucocorticoid receptor and 11 βHSD 1 and 2 mRNA levels.

## Centre(s)

Seventeen hospitals in the UK.

## Design

**Figure 1:**
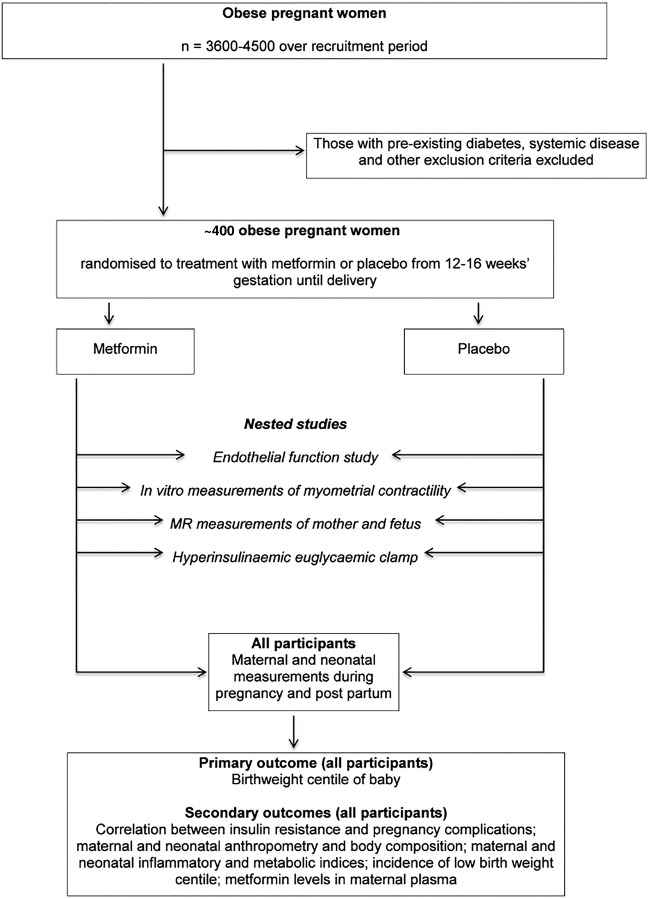
Flow chart of participants in the trial.

The design is a double-masked randomised placebo-controlled trial, with embedded substudies to explore mechanism of action of metformin, in a population of ∼400 obese pregnant women (figure 1).

## Inclusion and exclusion criteria

### Screening phase inclusion criteria

- Caucasian obese (BMI ≥30 kg/m^2^) pregnant women between 12^+0^ and 16^+0^ weeks’ gestation;
- Age greater than or equal to 16 years;
- Signed informed consent form.

### Screening phase exclusion criteria

- Non-Caucasian;
- BMI <30 kg/m^2^;
- Gestation >16^+0^ weeks;
- Pre-existing diabetes;
- Gestational diabetes in a previous pregnancy;
- Systemic disease at the time of trial entry, with the disease either requiring regular medication or having required treatment with systemic steroids in the past 3 months;
- Previous delivery of a baby <3rd centile by weight;
- Previous pregnancy complicated by pre-eclampsia prompting delivery before 32 weeks’ gestation;
- Known sensitivity to metformin hydrochloride or any of the excipients;
- Acute conditions at the time of trial entry with the potential to alter renal function such as dehydration sufficient to require intravenous infusion, severe infection, shock, intravascular administration of iodinated contrast agents;
- Acute or chronic diseases which may cause tissue hypoxia such as cardiac or respiratory failure, recent myocardial infarction, hepatic insufficiency, acute alcohol intoxication, alcoholism;
- Lactation;
- Multiple pregnancy.

### Randomisation exclusion criteria following screening

- Gestational diabetes mellitus (GDM) in index pregnancy (diagnosed with 75 g oral glucose tolerance test using WHO criteria, see table 1). Participants are also excluded if glucose tolerance test is diagnostic of GDM based on the criteria used in the recruiting centre.
- Known liver or renal failure or dysfunction at the time of trial entry tested prior to randomisation (defined as variable above the reference range shown in table 2).

**Table 1:** WHO diagnostic criteria for gestational diabetes following 75 g oral glucose tolerance test

Time (hours)	Blood glucose (mmol/L)
0	≥7.0
2	≥7.8

**Table 2:** Reference ranges for biochemical parameters

Variable	Range
Urea	2.5–6.6 mmol/L
Creatinine	<85 μmol/L
Sodium	135–145 mmol/L
Potassium	3.6–5.0 mmol/L
Bilirubin	3–16 μmol/L
ALT	10–60 IU/L

ALT, alanine transaminase.

### Ineligible and non-recruited participants

No further information will be collected on women who are ineligible solely because of abnormalities in glucose tolerance, liver or renal function, other than the number of such women for inclusion in trial metrics.

Telephone or face-to-face interviews will be carried out to explore the reasons for eligible women declining to participate.

## Methods

We will recruit women attending maternity hospitals in the UK. Women who fulfil all the potential eligibility criteria and who express an interest in the study will be provided with the participant information sheet and given at least 24 h to consider participation. They will then be asked to provide written informed consent. Following consent, participants will have screening blood tests including liver and renal function and a 75 g oral glucose tolerance test, to determine eligibility for randomisation. Participants will have demographics, medical history, height, weight and anthropometry measurements (waist, hips, mid-arm, mid-thigh circumference; tricep, bicep and subscapular skinfolds) recorded at this visit.

## Randomisation

Eligible participants will be randomly assigned to active treatment with metformin or an identical looking placebo. This will be documented in the patient's paper case record and/or computer file to show the woman's participation in the trial.

Participants will be randomised via a web portal connected to a central randomisation facility based at the trial data centre, the Edinburgh Clinical Trials Unit, University of Edinburgh. Baseline eligibility data will be required before randomisation. Participants will be randomised in a 1:1 ratio to metformin or placebo. Randomisation will be stratified by treatment centre and BMI 30–39 vs >40 kg/m^2^.

## Concealment of allocation

Randomising participants to active or placebo tablets will achieve concealment of allocation. Placebo tablets will appear identical to active treatment, so that the participant is masked to treatment allocation. The outcomes will be measured by clinicians and investigators masked to treatment allocation. Masking will not be broken until after data entry is complete, the validity of the data is checked, all queries resolved, the patient populations agreed and the database locked. Any clinically indicated emergency unmasking will be recorded prospectively.

## Intervention

Metformin tablets (or matched placebo) 500 mg administered from as soon as practicable after the point of randomisation (and certainly between 12 and 16 weeks’ gestation) until delivery of the baby. The dose regimen is as follows: week 1, 500 mg once daily; week 2, 500 mg twice a day; week 3, 500 mg three times a day; week 4, 500 mg morning and lunchtime and 1000 mg in the evening; week 5 until delivery of the baby, 1000 mg in the morning and evening and 500 mg at lunchtime. All doses are taken with food and dose escalation should continue to either the maximum tolerable dose or 2500 mg, whichever is higher.

### Dose changes

The local investigator or participant may alter the treatment regimen at his/her discretion, so long as the maximum daily dose does not exceed 2500 mg. Changes to the treatment dose will be recorded in the electronic case report form (eCRF) as soon as practicable.

### Other medications

Alcohol is prohibited due to the increased risk of lactic acidosis. Iodinated contrast agents may increase the risk of renal failure; hence, if they are required, metformin should be discontinued for at least 48 h from immediately prior to contrast agent administration until after renal function has been re-evaluated and found to be normal. Clinicians prescribing glucocorticoids (systemic and local routes), β2-adrenoreceptor agonists and ACE inhibitors should be aware that they might amplify or diminish the hypoglycaemic effect of metformin.

## Study assessments

Following randomisation, study assessments for glucose tolerance testing and other tests will occur as detailed in table 3.

**Table 3:** Summary of study visits

Visit number	1	2	3	4	5	6	7	8	9
Gestation	10–16 weeks	10–16 weeks	12–16 weeks	18–20 weeks	28 Weeks	36 Weeks	Term	Labour/delivery/neonatal	3 months postnatally
	Screening	Consent	Randomisation	Study visit (could be by telephone)	Study visit	Study visit	Study visit (could be by telephone)	Study visit	Study visit
Review inclusion and exclusion criteria	**X**								
Patient information leaflet	**X**								
Consent form		**X**							
Demographics		**X**							
Medical history		**X**							
Height and weight		**X**							**X**
Maternal anthropometry		**X**				**X**			**X**
Bloods for liver function/renal function/full lipid profile/CRP		**X**				**X**			
75 g OGTT (sampling at baseline and 2 hours)		**X**			**X**	**X**			
Stored sample for inflammatory and metabolic indices		**X**			**X**	**X**			
Randomisation			**X**						
Study drug dispensed			**X**		**X**				
Unused study drug/packaging returned								**X**	
Review SAEs				**X**	**X**	**X**	**X**	**X**	
Complete side effects questionnaire on eCRF				**X**	**X**	**X**	**X**	**X**	
Review and record pregnancy complications				**X**	**X**	**X**	**X**	**X**	
Saliva samples for cortisol measurements			X		X	X			
Bodpod measurements		X (OR VISIT 3)	X (OR VISIT 2)			X			X
Hyperinsulinaemic euglycaemic clamp						X			
FMD			X			X			
MR scan					X	X			
Labour/delivery information including birth weight, mode of delivery, EBL								X	
Cord blood and placenta biopsy								X	
Myometrium biopsy (if delivered by CS)								X	
Adipose tissue biopsy								X	
Baby's weight and anthropometry								X	X
Peapod measurements								X	X

CRP, C reactive protein; CS, caesarean section; EBL, estimated blood loss; eCRF, electronic case report form; FMD, flow mediated dilatation; OGTT, oral glucose tolerance test; SAE, serious adverse event.

Maternal anthropometric measurements recorded at baseline, 36 weeks’ gestation and 3 months postpartum, will include waist; hip; upper arm and thigh circumference and bicep, tricep and subscapular skinfold thickness. Neonatal anthropometric measurements recorded within 72 h of birth and at 3 months of age will include head circumference, and tricep and subscapular skinfold thickness. Length and weight will be recorded in order to calculate ponderal index.

Fasting maternal blood samples obtained at baseline, 28 and 36 weeks’ gestation will be stored for future analysis of inflammatory and metabolic indices, including (but not limited to) insulin, C reactive protein (CRP), interleukin (IL)-6, leptin, plasminogen activator inhibitor (PAI)1/PAI2 ratio, cortisol, lipids and non-esterified fatty acids. Cord blood obtained at the time of delivery will also be taken for measurement of glucose and stored for future measurement of the inflammatory and metabolic indices listed previously.

Table 3 also documents the timing of the nested substudies in which a subgroup of participants will participate. A summary of these is given below.

### Saliva samples

Diurnal cortisol levels will be measured in saliva samples collected at baseline, 28 and 36 weeks’ gestation. Saliva will be collected in salivettes at bedtime and on waking. Samples will be stored at −80°C. Cortisol will be measured by ELISA.

### Body composition

Maternal fat mass will be measured using air displacement plethysmography (BOD POD, http://www.lifemeasurement.com) at baseline, 36 weeks’ gestation and 3 months postpartum. Neonatal fat mass will be measured using the same technique (PEA POD, http://www.lifemeasurement.com) within 72 h of birth and at 3 months of age.

### Hyperinsulinaemic euglycaemic clamp

A subgroup of women will undergo a hyperinsulinaemic euglycaemic clamp study at 36 weeks’ gestation to characterise the relative effects of metformin on hepatic and peripheral insulin sensitivity.

### Flow-mediated dilatation

Endothelium-dependent flow-mediated dilatation will be measured in a subgroup of participants at baseline and again at 36 weeks’ gestation. Change in diameter of the brachial artery following a flow stimulus created by arterial occlusion will be measured using continuous two-dimensional grayscale ultrasound imaging.

### MRI and spectroscopy

A subgroup of participants will be scanned at 28 and 36 weeks’ gestation using a Verio 3 Tesla MRI system. T1-weighted acquisitions will be used to measure maternal subcutaneous and visceral fat; fetal liver volume; and fetal subcutaneous fat. Images will be analysed using the software program SliceOmatic. This program uses knowledge-based image processing to label pixels as fat or non-fat components and the adipose tissue mass derived using a mathematical model. Hepatic and skeletal muscle lipid content will be measured using ^1^H-magnetic resonance spectroscopy.

### Myometrial biopsies

A biopsy of the lower segment myometrium will be obtained from consenting participants who are delivered by caesarean section. The biopsies will be divided, with one portion placed in physiological saline for contractility studies and the other snap frozen for glycogen storage measurements.

## Participant compliance

Compliance will be recorded in a treatment diary and by counting of unused tablets. Additionally, gas chromatography mass spectrometry will be used to analyse metformin levels in blood samples from participants in the third trimester.

## Outcomes

### Primary outcome

Z-score corresponding to the gestational age-adjusted and sex-adjusted birthweight centiles of the baby.

### Secondary outcomes

- Maternal insulin resistance at 36 weeks’ gestation, which will be correlated with adverse pregnancy outcomes.
- Maternal anthropometry and body composition at 16 and 36 weeks’ gestation and 3 months postpartum.
- Baby anthropometry and body composition at birth and 3 months of age.
- Maternal inflammatory markers, lipid and fatty acid indices prior to starting treatment and again at 28 and 36 weeks’ gestation including CRP, IL-6, leptin, lipid profile, non-esterified fatty acids, polyunsaturated fatty acids and PAI1/PAI2 ratio.
- Neonatal CRP, glucose, insulin and other inflammatory and metabolic indices as previously described (measured in cord blood at birth).
- Incidence of low birthweight centile.
- Gas chromatography mass spectrometry measurement of metformin in maternal plasma to determine adherence.

Secondary outcomes from nested substudies;

- Maternal salivary cortisol levels at baseline, 28 and 36 weeks’ gestation.
- Hepatic and peripheral insulin sensitivity at 36 weeks’ gestation as measured by the hyperinsulinaemic euglycaemic clamp technique.
- Maternal brachial artery endothelium flow-mediated dilatation measured at 16 and 36 weeks’ gestation.
- Maternal subcutaneous and visceral adipose tissue deposition and hepatic and skeletal muscle ectopic fat deposition assessed using MRI and magnetic resonance spectroscopy.
- Fetal liver volume and fetal subcutaneous fat deposition assessed using MRI.
- In vivo measurements of myometrial contractility on myometrial biopsies obtained at the time of caesarean section.
- Placental glucocorticoid receptor and 11 βHSD 1 (β hydroxysteroid dehydrogenase 1) and 2 mRNA levels.

## Side effects and adverse events reporting

Participants are instructed to contact their investigator at any time after consenting to randomisation if any symptoms develop. In the case of any events, the investigator will initiate the appropriate treatment according to their medical judgement. Participants with adverse events (AEs) present at their last visit will be followed up until resolution of the event. All AEs and serious AEs (SAEs) that occur after randomisation will be recorded in detail in the participant's medical notes. SAEs occurring in the mother or baby from the time a participant is randomised until 30 days after stopping taking study treatment or until 28 days after delivery (whichever is later) will be reported to the co-sponsors using the trial documentation. The standard definition of a SAE will be used.^35^ For the purposes of this study the following events will not be considered SAEs: miscarriage; preterm labour; preterm prelabour spontaneous rupture of membranes; preterm delivery in the maternal interest; preterm delivery in the fetal interest; hospitalisation for pregnancy-induced hypertension; hospitalisation for maternal discomfort; hospitalisation for rest; hospitalisation for observation or monitoring for which the woman is admitted for a period of less than 12 h; delivery complications such as caesarean section or postpartum haemorrhage; admission of the baby to the neonatal unit for a period of up to 14 days.

## Statistical analysis plan, including sample size and power calculations

### Birthweight centiles

In a previous study, the mean (SD) birth weight in a cohort of obese women (mean BMI 34 kg/m^2^) was 4.0 (0.6) kg.^36^ We hypothesise that metformin will reduce mean birth weight by 0.2 kg, corresponding to a reduction in birthweight centile of 0.33 SD. We believe that this reduction is clinically relevant, but is a relatively conservative estimate of likely reduction in birthweight centile induced by metformin, given that mean birth weight in the study described above in a parallel non-obese cohort was 3.4 kg. A sample size of 143 in each group will have 80% power to detect a difference in mean birthweight centile of 0.33 SD (the difference between a placebo mean of 4.0 kg and a metformin mean of 3.8 kg) at the 5% significance level (2-sided) using a two group t test; a sample size of 163 in each group will give the study 85% power to detect these differences. In practice, we will recruit a larger sample size to allow loss to follow-up.

### Type of analysis and statistical tests

The primary analysis will be by intention to treat. Mean birthweight centile will be compared between the two groups using a two-sample t test, but with the analysis stratified for the same factors as the randomisation. Correlations within the metformin and placebo groups will be used to determine associations between insulin resistance and adverse pregnancy outcomes.

## Interim analysis and stopping rules

No formal interim analysis is planned (other than those requested by the Data Monitoring Committee (DMC)).

## Committee oversights

There is an independent Trial Steering Committee and an independent Data Monitoring Committee to oversee the safety of the participants in the trial.

## Discussion

Despite recognition that obesity represents a major public health problem, and that adult obesity may have its origins before birth, there is a lack of any effective intervention for obese pregnant women to improve pregnancy outcomes and reduce the future life risk of obesity for their offspring. In light of convincing evidence that the harmful effects of obesity during pregnancy are related to hyperglycaemia or insulin resistance, treatment during pregnancy with metformin is an exciting potential therapy. The Efficacy of Metformin in Pregnant Obese Women, a Randomised controlled (EMPOWaR) trial is designed to establish whether improving insulin sensitivity in pregnancy mitigates some of the adverse risk associated with obesity, with the primary aim of examining the effect on the birth weight of the baby, using birth weight as a surrogate marker of future life risk of obesity. A series of embedded mechanistic studies will help us to understand more about the mechanism of effect of metformin.

If our study finds metformin to be beneficial in reducing excess birth weight in obese pregnant women, it presents a potential future therapy where none currently exist. Clearly further large studies will be required to corroborate our findings. To our knowledge, there is currently only one other randomised double-masked placebo-controlled trial in progress which aims to recruit 850 women with a BMI >35 kg/m^2^ and is due to complete in September 2014 (Shehata, http://clinicaltrials.gov/show/NCT01273584).

As for any drug trial in pregnant women, safety is clearly a priority. Metformin has been used for decades in pregnant women with no evidence of any teratogenic effects. We do not expect any adverse effects from teratogenicity in our study population. There is no evidence from previous studies that metformin increases the incidence of babies with a low birthweight centile and we hope to confirm this with our data. Longer term follow-up studies of the offspring will be needed to assess the benefits or adverse effects in later life of antenatal exposure to metformin.

Finally, metformin is an inexpensive drug, costing under £5 per month. Obesity and the associated maternal and fetal complications are a huge financial burden on health services.^37^ If metformin were found to be effective, its use could contribute to significant financial savings for health services.

## Supplementary Material

Reviewer comments
