# Supplementary material for: Efficacy of metformin in pregnant obese women: a randomised controlled trial
Source: BMJ Open. 2015 Jan 14;5(1):e006854. doi: 10.1136/bmjopen-2014-006854 (PMC4298109; doi:10.1136/bmjopen-2014-006854)
Supplement: Reviewer comments [file bmjopen-2014-006854.reviewer_comments.pdf]

PEER REVIEW HISTORY

BMJ Open publishes all reviews undertaken for accepted manuscripts. Reviewers are asked to complete a checklist review form (**http://bmjopen.bmj.com/site/about/resources/checklist.pdf**) and are provided with free text boxes to elaborate on their assessment. These free text comments are reproduced below.

ARTICLE DETAILS

**TITLE (PROVISIONAL)**	Protocol: Efficacy of metformin in pregnant obese women, a randomised controlled trial
**AUTHORS**	Chiswick, Carolyn; Reynolds, Rebecca; Denison, Fiona; Whyte, Sonia; Drake, Amanda; Newby, David; Walker, Brian; Forbes, Shareen; Murray, Gordon; Quenby, Siobhan; Wray, Susan; Norman, Jane

This article was not externally reviewed at BMJ Open. Protocols that have been independently assessed prior to submission to BMJ Open are usually fast-tracked to publication on the grounds that further substantial changes will not be possible. This independent assessment will usually be external, independent review for both a substantial grant award from a non-commercial or government funder and ethics approval.
